# A tissue-targeted prime/pull/keep therapeutic herpes simplex virus vaccine protects against recurrent ocular herpes infection and disease in HLA-A*0201 transgenic rabbits

**DOI:** 10.1128/jvi.00135-25

**Published:** 2025-04-10

**Authors:** Aziz A. Chentoufi, Swayam Prakash, Hawa Vahed, Sweta Karan, Afshana Quadiri, Anthony B. Nesburn, Lbachir BenMohamed

**Affiliations:** 1 Laboratory of Cellular and Molecular Immunology, Gavin Herbert Eye Institute, University of California Irvine, School of Medicine 12219, Irvine, California, USA; 2 Department of Molecular Biology & Biochemistry, University of California Irvine, School of Medicine 209685, Irvine, California, USA; 3 Institute for Immunology, University of California Irvine, School of Medicine 12219, Irvine, California, USA; Lerner Research Institute, Cleveland Clinic, Cleveland, Ohio, USA

**Keywords:** HSV-1, ocular, herpes, HLA transgenic rabbit, CD8^+^ T cell, prime/pull/keep vaccine

## Abstract

Herpes simplex virus type 1 (HSV-1) continues to be one of the most prevalent viral infections globally, with approximately 3.72 billion individuals affected worldwide. A clinical herpes vaccine is still lacking. In the present study, a novel prime/pull/keep vaccine was tested in a human leukocyte antigen transgenic rabbit model of ocular herpes (HLA-A*0201 Tg rabbit). Ten asymptomatic (ASYMP) CD8^+^ T-cell peptide epitopes and 3 CD4^+^ T-cell epitopes were selected from the HSV-1 glycoproteins D and B (gD and gB), viral tegument proteins (VP11/12 and VP13/14), and the DNA replication-binding helicase (UL9), all preferentially recognized by CD8^+^ and CD4^+^ T cells from “naturally protected” HSV-1-seropositive healthy ASYMP individuals (who never had recurrent corneal herpetic disease). HLA Tg rabbits were ocularly infected with HSV-1, then during latency at day 30 post-infection, the rabbits were ocularly vaccinated with a recombinant neurotropic AAV8 vector (10^7^GC/ eye) encoding for the 10 CD8^+^ T-cell peptide and 4 CD4^+^ T-cell peptide (prime), T-cell attracting CXCL-11 (pull), and T-cell keeping IL-2/IL-15 cytokines (keep). The rabbits were followed up for corneal disease and viral loads in tears for 28 days. The frequency, function, and protective efficacy of HSV-specific CD8^+^ T cells induced by the prime/pull/keep vaccine were assessed in the trigeminal ganglia (TG), cornea, spleen, and peripheral blood. Compared to the mock group (unvaccinated), the peptides/CXCL11/IL-2/IL-15 vaccine generated frequent resident CD8^+^ T cells that infiltrated the TG. In ocularly HSV-1-infected and prime/pull/keep vaccinated rabbits, CD8^+^ T cell mobilization and retention into TG were associated with a significant reduction in corneal herpes infection and disease. These findings draw attention to the novel prime/pull/keep therapeutic vaccine strategy to mobilize and retain antiviral T cells to tissues protecting them against herpetic infection and disease.

**IMPORTANCE:**

There is an urgent need for a vaccine against widespread human herpes simplex virus infections. The present study demonstrates that immunization of humanized HLA-A*0201 transgenic rabbits with CD8^+^ and CD4^+^ T-cell epitope peptides (prime)/ CXCL11 (pull)/ IL-2/IL-15 (keep) AAV8-based vaccine triggered mobilization and retention of HSV-1-specific CD8^+^ T cells locally in the cornea and TG, the sites of acute and latent herpes infections. Mobilization and retention of antiviral CD8^+^ T cells into the cornea and TG of HSV-1-infected rabbits that received the prime/pull/keep vaccine was associated with protection against ocular herpes infection and disease. These results highlight the importance of the prime/pull/keep vaccine strategy to bolster the number and function of protective CD8^+^ T cells within infected tissues.

## INTRODUCTION

Herpes simplex virus type 1 (HSV-1) continues to be one of the most prevalent viral infections globally, with approximately 3.72 billion individuals affected worldwide (1, 2). HSV-1-associated ocular herpetic infection ranges from asymptomatic to symptomatic clinical conditions including blepharitis, conjunctivitis, neovascularization, disciform stromal edema, and the vision-threatening condition herpetic stromal keratitis (HSK) (3–10). Despite the role of the corneal epithelium in the first phases of ocular herpes infection, the mechanisms by which HSV-1 enters human corneal epithelial cells are poorly understood. Agelidis and Shukla have eloquently summarized what has been known in recent years about the mechanisms of HSV cellular entry in the cornea (11).

More than 450,000 individuals in the United States alone have suffered from blinding ocular herpetic disease, necessitating medical intervention such as antiviral drug treatment and corneal transplantation (12–14). Despite current antiviral therapies, such as Acyclovir and its derivatives that merely reduce the symptoms of herpetic disease by about 45% (3), HSV-1 ocular infection continues to rise globally, and the development of an effective vaccine would represent a critical advancement, offering a more sustainable and cost-effective method to combat corneal herpetic infections and disease (reviewed in reference 15).

Evidence from human cadaveric brain studies (15–17) has shown that CD8^+^ T cells residing in both the trigeminal ganglia (TG) and cornea play an essential role in controlling the reactivation of latent HSV-1 from sensory neurons, thereby preventing corneal herpetic infections and disease (18, 19). However, herpes vaccine clinical trials, which employed recombinant glycoproteins B and D (gB and gD), failed to protect humans from herpetic disease despite generating high levels of HSV-specific neutralizing antibodies (20, 21). These disappointing results highlight the importance of inducing strong T-cell responses, in addition to antibody-mediated immunity, to achieve protective immunity against ocular herpes. For an effective T-cell-mediated immunity against viral pathogens, it is fundamentally important for these T cells to migrate to the site of infection and for an optimal number of memory resident T cells to be established.

The migration of HSV-specific CD8^+^ T cells to key anatomical sites of latent infection, such as the TG, is thought to be tightly regulated by interactions between chemokines and their corresponding chemokine receptors, a process that can be influenced by the vaccine strategy employed (22–26). Increasing evidence supports the notion that CD8^+^ T cells specific to HSV epitopes possess intrinsic characteristics that guide their migration to the cornea and TG, which are the primary sites of acute and latent HSV-1 infections, respectively. However, the TG is recognized as an immunologically restrictive environment, meaning it is not easily accessible to CD8^+^ T cells that are activated by a conventional parenteral vaccine and subsequently enter the circulation (27). This is thought to be due to low levels of T-cell-attracting chemokines, such as CXCL9, CXCL10, CXCL11, and CCL5, in non-infected TG tissue, which limits the migration of sufficient antiviral CD8^+^ T cells from the bloodstream into the TG.

In this study, we proposed that a prime/pull/keep AAV8-based vaccine strategy, designed to both “prime” functional antiviral CD8^+^ T cells in peripheral tissues and “pull” and “keep” them in the infected cornea and TG, would lead to a significant reduction in corneal HSV-1 infection and disease. To evaluate this hypothesis, we utilized the recently developed “humanized” HLA-A*0201 transgenic rabbits (HLA Tg rabbits), which represent the most reliable small animal model for studying ocular herpes infection and associated diseases (18, 19, 28–30). Our results showed that immunizing HLA-A*0201 Tg rabbits with a topical ocular application of a recombinant AAV8 vector carrying HSV-1 CD8^+^ and CD4^+^ T-cell epitopes, along with the chemokine CXCL-11 and cytokines IL-2/IL-15, led to (i) strong activation of HSV-specific CD8^+^ T cells in the trigeminal ganglia (TG), with a resident memory T-cell phenotype and (ii) increased local recruitment of CD8^+^ T cells to the TG. Importantly, this enhanced immune response was associated with a significant reduction in ocular HSV-1 infection and milder recurrent herpetic corneal disease.

These preclinical findings in the HLA-A*0201 Tg rabbit model, a well-established model of ocular herpes infection, strongly suggest that the prime/pull/keep vaccine strategy—based on HSV-1 human epitope peptides combined with CXCL11 and IL-2/IL-15 cytokines—has the potential to provide robust, safe, and protective immunity. This approach should be considered in the future development of a clinical vaccine for ocular herpes, as it offers a promising solution for controlling both HSV-1 infection and herpetic corneal disease.

## MATERIALS AND METHODS

### HLA-A*02:01 transgenic rabbits

HLA Tg rabbits were derived from New Zealand White rabbits (31). The HLA Tg rabbits retain their endogenous rabbit MHC locus and express human HLA-A*0201 under the control of its normal promoter (32, 33). Prior to this study, the expression of HLA-A*0201 molecules on the PBMC of each HLA Tg rabbit was confirmed by FACS analysis. In brief, PBMCs were stained with 2 µL anti–HLA-A2 mAb, BB7.2 (BD Pharmingen, San Diego, CA), at 4°C for 30 min, washed, and analyzed by flow cytometry using an LSRII (Becton Dickinson, Mountain View, CA). The acquired data were analyzed with FlowJo software (TreeStar, Ashland, OR). All rabbits used in these studies had a similarly high level of HLA-A*0201 expression (>90%). This eliminated any potential bias due to the variability of HLA-A*0201 molecule levels in different animals. New Zealand White rabbits (non-Tg control rabbits), purchased from Western Oregon Rabbit Co., were used as controls (7, 32, 34, 35). All rabbits were housed and treated in accordance with ARVO (Association for Research in Vision and Ophthalmology), AAALAC; Association for Assessment and Accreditation of Laboratory Animal Care, and NIH (National Institutes of Health) guidelines.

### Peptide synthesis

CD4^+^ and CD8^+^ T-cell epitope peptides from the HSV-1 glycoproteins D and B (gD and gB), viral tegument proteins (VP11/12 and VP13/14), and the DNA replication binding helicase (UL9) proteins were synthesized by 21st Century Biochemicals (Marlboro, MA). All peptides (CD8^+^ T-cell epitopes: *gD_53-61_, gD_278-296_, gB_183-191_, gB_342-350_, gB_561-569_, VP11_220-228_, VP11_702-710_, VP13/14_504-512_, VP13/14_544-552_, UL9_196-204_*; CD4^+^ T-cell epitopes: *gB_166-180_, VP11_129-143_, gD_49-82_*) were HPLC purified to a purity of 95% to 98% as determined by reversed-phase high-performance liquid chromatography (Vydac C18) and mass spectroscopy (Voyager MALDI-TOF System). Stock solutions were made at 1 mg/mL in phosphate-buffered saline (PBS). All peptides were aliquoted and stored at −20°C until assayed.

### Design and construction of AAV8 vectors expressing CD8^+^ and CD4^+^ T-cell epitopes, CXCL11 chemokine, and IL-2/IL-15 under neurotropic CamKIIα promoters

**Fig 1:**
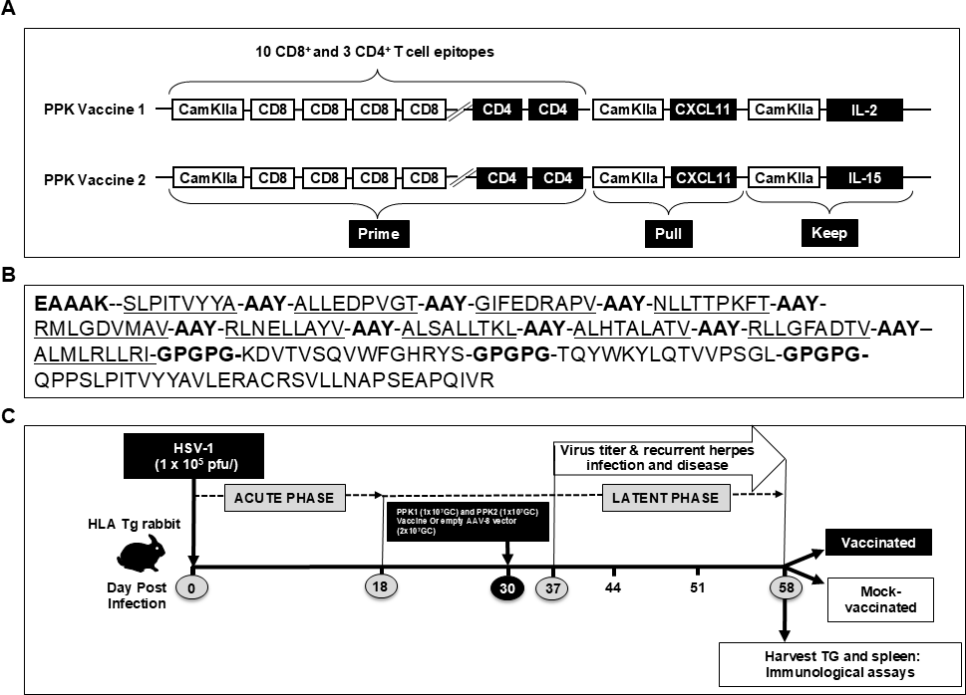
Prime-pull-keep vaccine construct design and strategies for vaccination. (A) Illustration shows prototype of the two prime-pull-keep (PPK) vaccine approaches. The first approach PPK1 contains a multi-epitope herpes vaccine consisting of immunogenic 10 CD8^+^ T-cell epitopes and 3 CD4^+^ T-cell epitopes meant to prime the T cells; CXCL11 molecule to pull the T cells; and IL-2 to keep the T cells to protect against herpes simplex virus infection. Whereas the second approach shows vaccine PPK2 contains a multi-epitope herpes vaccine consisting of the same immunogenic 10 CD8^+^ T-cell epitopes and 3 CD4^+^ T-cell epitopes as PPK1 meant to prime the T cells; CXCL11 molecule to pull the T cells; and IL-15 to keep the T cells to protect against herpes simplex virus infection. (B) Details of amino acid sequences of 10 CD8^+^ T-cell epitopes and 3 CD4^+^ T-cell epitopes and the linkers (Bold text) used in between for these constructs that have been selected and tested as previously described (7, 32, 34, 35). (C) Schematic representation of the experimental design where HLA Transgenic rabbits were having HSV-1 (strain McKrae) ocular infection (1 × 10^5^ PFU/eye) followed by PPK immunization. Rabbits were followed for an acute phase of HSV-1 infection for 18 days. Subsequently, 30 days post-HSV-1 infection, the transgenic rabbits were immunized with adenovirus-associated virus-8 (AAV8)-based PPK vaccines at 1 × 10^7^ GC. Immunological, virological, and eye disease were monitored during the latent phase of herpes infection between day 37 and day 58 post-HSV-1 infection. On Day 58 post-infection, the HLA-A*0201 Tg rabbits were euthanized, and Trigeminal ganglia and cornea were collected from vaccinated and mock-vaccinated groups for immunological and virological assays.

Two prototype PPK vaccine molecules were used: (i) AAV8-vector expressing 10 CD8^+^ and 3 CD4^+^ T-cell epitopes together with CXCL11 and IL-2 and (ii) AAV8-vector expressing the same 10 CD8^+^ and 3 CD4^+^ T-cell epitopes together with CXCL11 and IL-15. Both candidate vaccines expressed the same 10 CD8^+^ T-cell epitopes and 3 CD4^+^ T helper epitopes. The CD8^+^ T-cell epitopes were separated by the linker AAY and the CD4^+^ T-cell epitopes were separated by the linker GPGPG as described in Fig. 1B and our previous publications (7, 32, 34, 35). The PPK vaccines were made by vector Biolabs, Inc., where rabbit CXCL11 and IL-2 or IL-15 were co-expressed by a neurotropic non-replicating AAV8 vector in tandem with the CD8^+^ and CD4^+^ T-cell epitopes, under the control of the CamKIIα neuron-specific promoter (Fig. 1A).

### Prime/pull/keep vaccination after ocular herpes challenge

HLA-A*0201 Tg rabbits (8–10 weeks) with similar expression levels of HLA-A*02:01 molecules (> 90%) were used, as described below. Age-matched HLA-A*02:01 rabbits (*n* = 12) were ocularly infected with HSV-1 (1 × 10^5^ PFU). On day 30 post-infection with HSV-1, only 10 rabbits survived. Five rabbits were treated with empty AAV8 vector (mock-vaccinated), and the other five rabbits received topical ocular treatment with 1 × 10^7^ viral genome copies (GC) of a recombinant rAAV8-CamKIIa-NP7-CamKIIa-CXCL11-CamKIIa-IL-2 vector and 1 × 10^7^ GC of a recombinant rAAV8-CamKIIa-NP7-CamKIIa-CXCL11-CamKIIa-IL-15 vector (combined vectors administration constitute PPK vaccine).

### Herpes simplex virus production

HSV-1 (strain McKrae) was used in this study. To create large quantities of HSV-1 stock, we routinely purified HSV-1 (strain McKrae) through plaque assay three times (triple plaque-purified assay). The parental McKrae viruses were triple plaque purified and passaged only one or two times prior to use as previously described (23, 36) using rabbit skin cell monolayers grown in MEM (1×) containing 10% FBS, 2 mM L-glutamine, 2.5 µg/mL amphotericin, and 5% penicillin-streptomycin solution (from a stock of 10,000 IU penicillin and 10,000 µg/mL streptomycin).

### Ocular infection of rabbits with HSV-1

Without making any corneal scarification, rabbits were ocularly infected (both eyes) by dropping 5 µL HSV-1 (1 × 10^5^ PFU) strain McKrae suspended in culture medium on day 0. We have chosen the McKrae strain over other HSV-1 strains such as KOS that have been shown to need corneal scarification and have poor reactivation phenotype and recurrent eye disease in mouse and rabbit models (37).

### Rabbit corneal disease clinical scores

Rabbits were examined for ocular disease and viral loads for 28 days after the vaccination with the PPK or mock constructs. The ocular disease was determined by masked investigators (four lab members and the principal investigator) using fluorescein staining and slit lamp examination before vaccination on day 30 and days 37, 44, 51, and 58 thereafter. The corneal lesion size was expressed as the area of staining in square millimeters using the Image J program (version 1.54g; National Institutes of Health). These examinations were performed by investigators blinded to the treatment regimen of the rabbits.

### Quantification of ocular infectious virus

Tears were collected from both eyes using a Dacron swab (type 1; Spectrum Laboratories, Los Angeles, CA) on days 7, 14, 21, and 28 post-vaccination. Individual swabs were transferred to a 2 mL sterile cryogenic vial containing 500 µL culture medium and stored at −80°C until use. The HSV-1 DNA loads in tear samples were determined by standard real-time PCR based on previously described reaction conditions (38).

### Preparation of rabbit cell suspensions from TG and spleen

Rabbits were euthanized, and TG and spleen were isolated and finely minced using dissection scissors. Then digested in DMEM–5% fetal bovine serum (FBS) containing collagenase I (Life Technologies, Carlsbad, CA), as we previously described (5, 15, 17, 37). All samples were placed at 37°C in a shaker at 250 rpm and incubated for 1 h (TG) and 15 min (spleen) and passed through a 70 µm nylon cell strainer followed by a 40 µm nylon cell strainer. The cell suspensions were centrifuged and resuspended in complete media, then lymphocyte populations were isolated on a Percoll gradient of 40%. Cell suspensions were spun down at 1,400 rpm for 5 min at 4°C and then washed and suspended in fluorescence-activated cell sorter (FACS) buffer (PBS–0.01% NaN3–0.1% bovine serum albumin [BSA], 2 mM EDTA) for FACS acquisition and analysis.

### Rabbit peripheral blood mononuclear cell isolation

10 mL of blood was drawn from each rabbit into yellow-top Vacutainer Tubes (Becton Dickinson, USA). Sera were isolated by centrifugation for 10 min at 800 *g*. PBMCs were isolated by gradient centrifugation using a leukocyte separation medium (Cellgro, USA). The cells were washed in PBS and re-suspended in complete culture medium consisting of RPMI-1640 medium containing 10% FBS (Bio-Products, Woodland, CA, USA).

### Flow cytometry assays on rabbit T cells

Cell suspensions from TG, spleen, and blood were analyzed by flow cytometry after staining with HLA-tetramers-PE and panels of anti-rabbit CD8, CD4, CD69, CD62L, CD103, and CD44. The following anti-rabbit antibodies were used: anti-CD8 (clone MCA1576F, Serotec) FITC, -CD4 (clone KEN-4) FITC, -CD44 (clone IM7) PE-Cy7, -CD62L (clone DREG-56) Alexa Fluor 700, -CD69 (clone FN50) APC/Cy7, -CD103 (clone LF61) PercP.Cy5.5. For surface stain, panels of mAbs against various cell markers were added to a total of 1 × 10^6^ cells in 1× PBS containing 1% FBS and 0.1% sodium azide (FACS buffer) for 45 min at 4°C. Cells were washed again with Perm/Wash and FACS Buffer and fixed in PBS containing 2% paraformaldehyde (Sigma-Aldrich, St. Louis, MO). For each sample, 500,000 total events were acquired on the BD LSRII. Ab capture beads (BD Biosciences) were used as individual compensation tubes for each fluorophore in the experiment. To define positive and negative populations, we employed fluorescence minus controls for each fluorophore used in this study when initially developing staining protocols. In addition, we further optimized gating by examining known negative cell populations for background-level expression.

### Rabbit CD8^+^ T-cell tetramer assays

**Fig 2:**
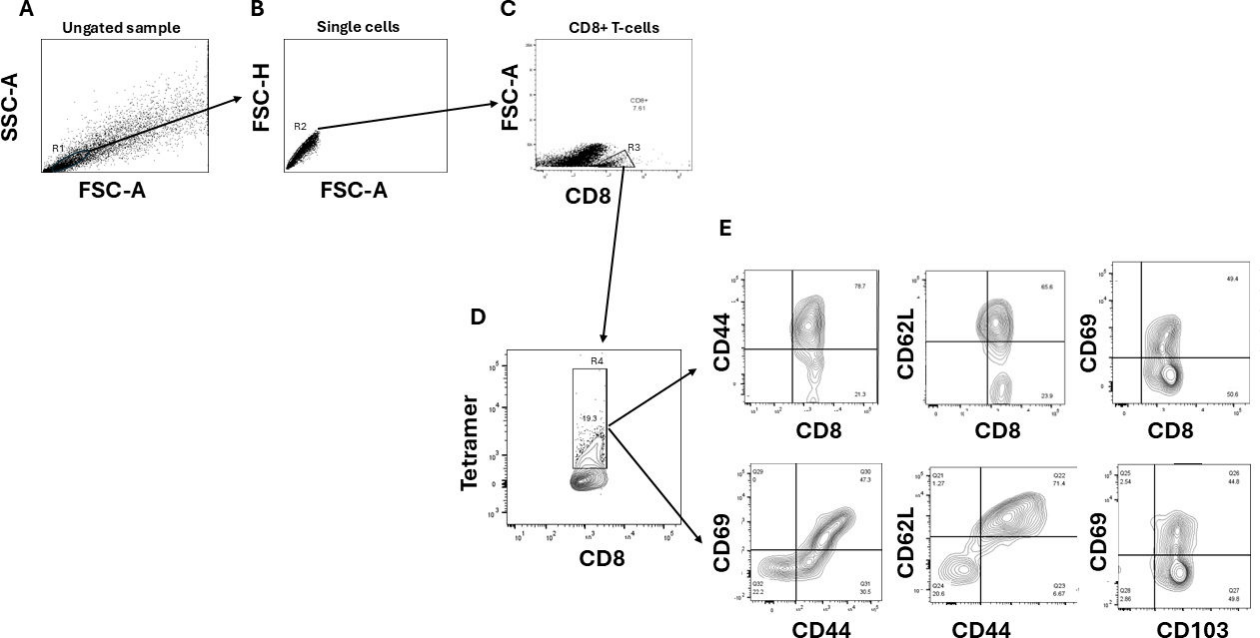
Example of flow cytometry gating strategy for HSV-1-specific CD8^+^ T cells in the TG. TG cell suspensions were stained with two distinct panels of antibodies: one for CD8^+^ T cells and one for CD4^+^ T cells. The cell population was first plotted on an ungated plot showing size and granularity (A), then a first gate (R1) was performed on lymphocyte area and plotted as single cells (B); then a second gate (R2) was performed and the cell population was re-plotted based on CD8 expression (C) then a third gate (R3) was performed on CD8^+^ T cells and plotted on tetramer-positive population (D) showing the HSV-specific CD8^+^ T cells. To study the frequencies of memory CD8^+^ TCM, CD8^+^ TEM, and CD8^+^ TRM cell subsets detected by FACS, we have created a fourth gate (R4) and we plotted the positive populations in different plots such as CD8/CD44, CD8/CD52L, CD8/CD69, CD44/CD69, CD44/CD62L, and CD103/CD69 (E).

For tetramer-specific CD8^+^ T-cell frequencies, TG, spleen, and PBMCs were analyzed for the frequency of CD8^+^ T cells specific to each of the 10 CD8^+^ T-cell epitopes using the corresponding HLA-A2-peptide/Tetramer, provided by the NIH tetramer facility (7, 39). A human beta-2-microglobulin was incorporated in the tetramers, as no rabbit beta-2-microglobulins are currently available. Briefly, the cells were first incubated with 1 µg/mL of each of the three PE-labeled HLA-A2-peptide/Tetramer at 37°C for 30–45 min. The cells were washed twice and stained with panels of anti-Rabbit CD8, CD4, CD62L, CD69, CD103, and CD44. After two additional washings, cells were fixed with 2% formaldehyde in FACS buffer. A total of 500,000 events were acquired by LSRII followed by analysis using FlowJo software, and functional memory HSV-1-specific T cells were gated as described in Fig. 2. The absolute numbers of individual peptide-specific CD8**^+^** T cells were calculated using the following formula: (no. of events in CD8^+^/Tetramer^+^ cells) × (no. of events in gated lymphocytes)/(no. of total events acquired).

### Rabbit TG and cornea histopathology

Rabbits were euthanized; corneas and TG were harvested, embedded in Tissue-Tek (OCT compound; VWR International, West Chester, PA), and snap-frozen. Approximately 7 μm-thick cryosections were fixed in acetone and stored at −80°C. Sections were H&E stained and mounted. CD4^+^ and CD8^+^ T cell infiltration in the TG and cornea were examined using a Keyence BZ-X700 microscope at 10× magnification and imaged using z-stack.

### Statistical analyses:

Data for each assay were compared by analysis of variance (ANOVA) and Student’s *t*-test using GraphPad Prism version 5 (La Jolla, CA). Differences between the two groups were identified by ANOVA and multiple comparison procedures, as we previously described (39). Data are expressed as the mean ± SD. Results were considered statistically significant at *P* < 0.05.

## RESULTS

### A multi-epitope/CXCL11/IL-2/IL-15 (prime/pull/keep) vaccine design

Ten CD8^+^ T-cell epitope peptides that exhibited high affinities for soluble HLA-A*0201 and 3 CD4^+^ T-cell epitopes from 5 HSV-1 proteins were selected for the design of the PPK vaccine (Table 1). We sought to determine whether priming with the mixture of these HSV-1 peptides expressed by AAV-8 encoding for CXCL11 (pull) and IL-2 or IL-15 (keep) would (i) boost the protective efficacy of HSV-1 epitope-specific CD8^+^ T cells and (ii) pull more HSV-1-specific CD8^+^ T cells within the TG and cornea, the sites of acute and latent HSV-1 infection in rabbits. Using the optimal CamKIIαpromoter, we constructed two neurotropic recombinant non-replicating adeno-associated virus type 8 vector (rAAV8) expressing the CD8^+^ and CD4^+^-specific T-cell epitope peptides, T-cell-attracting CXCL11 chemokine and IL-2 or IL-15: rAAV8-multi-epitope-CamKIIα-CXCL11-CamKIIα-IL-2 and rAAV8-multi-epitope-CamKIIα-CXCL11-CamKIIα-IL-15 (PPK1 and PPK2) (Fig. 1A). Both candidate vaccines PPK1 and PPK2 expressed the same 10 CD8^+^ T-cell epitopes and 3 CD4^+^ T helper epitopes. The CD8^+^ T-cell epitopes were separated by the linker AAY and the CD4^+^ T-cell epitopes were separated by the linker GPGPG as described in Fig. 1B.

**TABLE 1:** CD4^+^ and CD8^+^ T-cell epitope peptides position and amino acid sequences from HSV-1 glycoproteins D and B (gD and gB), viral tegument proteins (VP11/12 and VP13/14), and the DNA replication-binding helicase (UL9) proteins

Peptide position	CD8^+^ T-cell epitope	Peptide position	CD4^**+**^ T-cell epitope
*gD_53-61_*	SLPITVYYA	*gB_166-180_*	KDVTVSQVWFGHRYS
*gD_278-296_*	ALLEDPVGT	*VP11/12_129-143_*	TQYWKYLQTVVPSGL
*gB_183-191_*	GIFEDRAPV	*gD_49-82_*	QPPSLPITVYYAVLERACRSVLLNAPSEAPQIVR
*gB_342-350_*	NLLTTPKFT		
*gB_561-569_*	RMLGDVMAV		
*VP11/12_220-228_*	RLNELLAYV		
*VP11/12_702-710_*	ALSALLTKL		
*VP13/14_504-512_*	ALHTALATV		
*VP13/14_544-552_*	RLLGFADTV		
*UL9_196-204_*	ALMLRLLRI		

### Human leukocyte antigen (HLA-A*0201) transgenic rabbits used for pre-clinical evaluation of a multi-epitope/CXCL11/IL-2/IL-15 (prime/pull/keep) vaccine against recurrent ocular herpes infection

**Fig 3:**
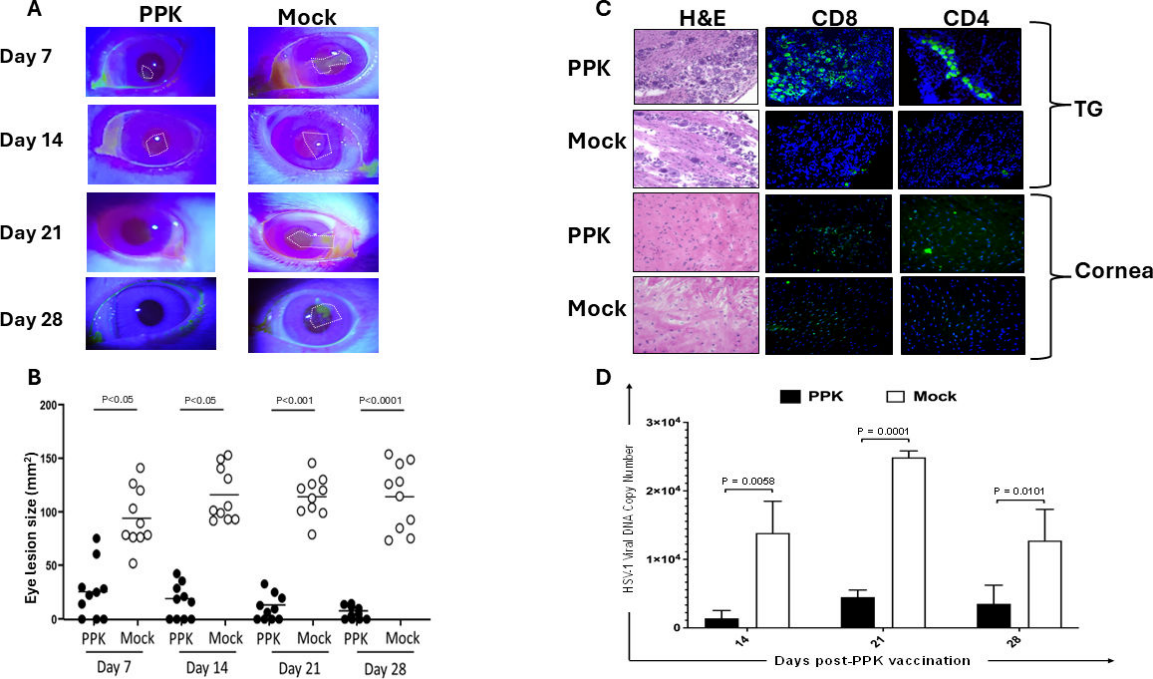
Physical estimation, histopathological evaluation, and HSV-1 viral DNA copy number showing reduced disease pathology in HLA-A*0201 transgenic rabbits vaccinated with PPK vaccine in comparison to mock-vaccinated rabbits. (A) Representative images showing reduced recurrent corneal herpetic disease in HLA-A*0201 Tg rabbits vaccinated with PPK vaccine in comparison to mock-vaccinated HLA-A*0201 Tg rabbits. The fluorescein staining is outlined in the figure and serves to measure the size of the corneal lesions. (B) Scatter graph showing eye lesion size (mm^2^) among PPK-vaccinated and mock-vaccinated HLA transgenic rabbits at days 7, 14, 21, and 28 post-vaccinations. The corneal lesion size was expressed as the area of staining in square millimeters using the Image J program (version 1.54g; National Institutes of Health). (C) Representative H & E, CD8, and CD4 staining images of the trigeminal ganglia and cornea at day 58 p.i. with HSV-1 infection in PPK-vaccinated vs. Mock-vaccinated HLA-A*0201 Tg rabbits. Images were taken at 10× magnification. (D) Bar diagrams showing a comparison of HSV-1 viral loads in the eyes of PPK-vaccinated vs. Mock-vaccinated HLA-A*0201 transgenic rabbits. Viral genome load was quantified by PCR from eye swabs of PPK-vaccinated and Mock-vaccinated HLA Tg rabbits. The data are representative of one independent experiment, and the graphed values and bars represent the SD between the two experiments. The difference between the groups is significant when the *P*-value is <0.005.”

HLA-A*0201-positive transgenic rabbit breeders were selected based on their high expression of HLA-A*0201 molecules since the expression of the rabbits’ own MHC class I molecules might interfere with the human HLA-A*0201-restricted responses (Materials and Methods). The high expression of HLA-A*0201 molecules in the selected HLA Tg rabbits should result in rabbit CD8^+^ T cells using the human HLA-A*0201 molecules both at the thymus selection and peripheral effector levels (30). All selected HLA Tg rabbits had a similar high expression of HLA-A*0201 molecules in over 95% of PBMC, as previously described (7, 32, 35). HLA Tg rabbits (*n* = 12) were ocularly infected with HSV-1 (McKrae, 1 × 10^5^ PFU/eye) at day 30 post-infection, 10 rabbits survived, and 5 rabbits were vaccinated with the PPK1 (1 × 10^7^ GC) and PPK2 (1 × 10^7^ GC) vaccines, as shown in Fig. 1C and five HLA Tg rabbits were mock-vaccinated and received an empty AAV8 vector (2 × 10^7^ GC), as control (Mock). Rabbits were followed up for corneal eye disease and viral loads in the tears for 30 days and then sacrificed at day 58 post-infection. The PPK-treated group displayed significantly less recurrent corneal herpetic disease (Fig. 2A and B) and virus loads in the eyes (Fig. 3D). By contrast, the mock non-vaccinated group showed a significantly higher level of virus replication in eyes associated with severe recurrent ocular herpetic disease (Fig. 2A, B and D). Histopathological analysis (Fig. 3C) shows that PPK-immunized rabbits have a higher number of mononuclear cells including CD4^+^ and CD8^+^ T cells in the TGs and the cornea, suggesting that the immunization with PPK induced a higher number of CD4^+^ and CD8^+^ T cells in the site of HSV-1 infection (cornea and TGs).

### A multi-epitope/CXCL11/IL-2/IL-15 (prime/pull/keep) vaccine bolsters the frequencies of resident memory CD8^+^ TEM, TCM, and TRM T cells in HLA transgenic rabbits

**Fig 4:**
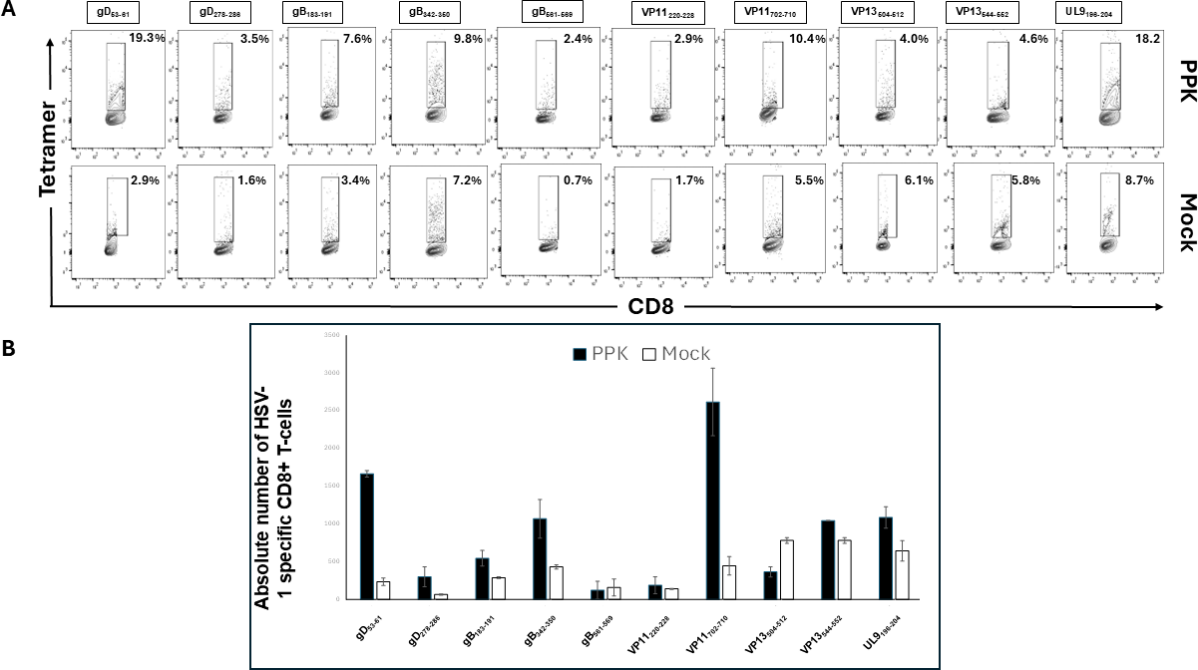
Higher magnitude of HSV-1-specific CD8^+^ T cells observed in the Trigeminal ganglia of PPK*-*vaccinated HLA-A*0201 transgenic rabbits. (A) Flow cytometry analysis of the frequencies of CD8^+^ T cells specific to HLA-A*0201-restricted HSV-1 epitopes (gD53-61, gD278-286, gB183-191, gB342-350, gB561-569, VP11220-228, VP11702-710, VP13504-512, VP13544-552, and UL9196-204) in TG of PPK-vaccinated and Mock-vaccinated HLA-Tg rabbits. Higher frequencies of CD8^+^ T cells specific to HLA-A*0201-restricted HSV-1 gD_53-61_, gB_342-350_, VP11,_702-710,_ and UL9_196-204_ epitopes detected by Tetramer staining in TG of PPK vaccinated HLA-Tg rabbits compared to mock-vaccinated rabbits. (B) Corresponding bar diagrams showing an absolute number of HSV-1-specific CD8^+^ T cells in the TG of PPK-vaccinated and Mock-vaccinated HLA-Tg rabbits. The data are representative of two independent experiments, and the graphed values and bars represent the SD between the two experiments. Overall, the difference between the two groups for the absolute number of HSV-1 epitope-specific CD8^+^ T cells is significant (*P* < 0.005) except for HSV-1 gD_561-569_, VP_220-228_ epitope-specific T cells.

**Fig 5:**
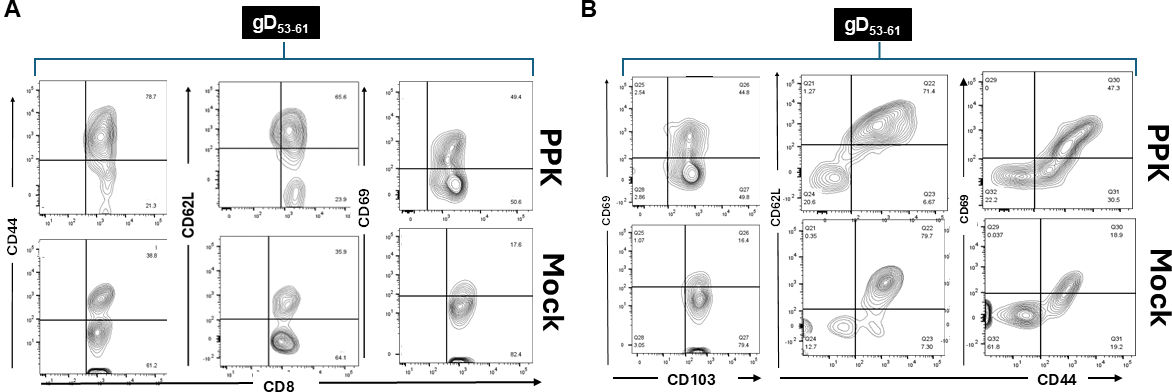
Increased frequencies of CD8^+^ memory T-cell population observed in response to prime-pull-keep vaccination approach. (A and B) Representative flow cytometry data showing the frequencies of HSV-1 gD_53-61_-specific memory CD8^+^ T_CM_, CD8^+^ T_EM_, and CD8^+^ T_RM_ cell subsets detected in HSV-1 infected TG of PPK-vaccinated and Mock-vaccinated HLA Tg rabbits. The data are representative of one immunodominant epitope HSV-1 gD_53-61_ inducing a high frequency of memory CD8^+^ T_CM_, CD8^+^ T_EM_, and CD8^+^ T_RM_ cells. Similar data were obtained with the three other immunodominant epitopes tested (gB_342-350_, VP11_702-710_, and UL9_196-204_). To study the CD8^+^ T-cell memory response in the context of the PPK vaccination approach, multiple markers like CD44, CD69, CD62L, and CD103 were analyzed.

**Fig 6:**
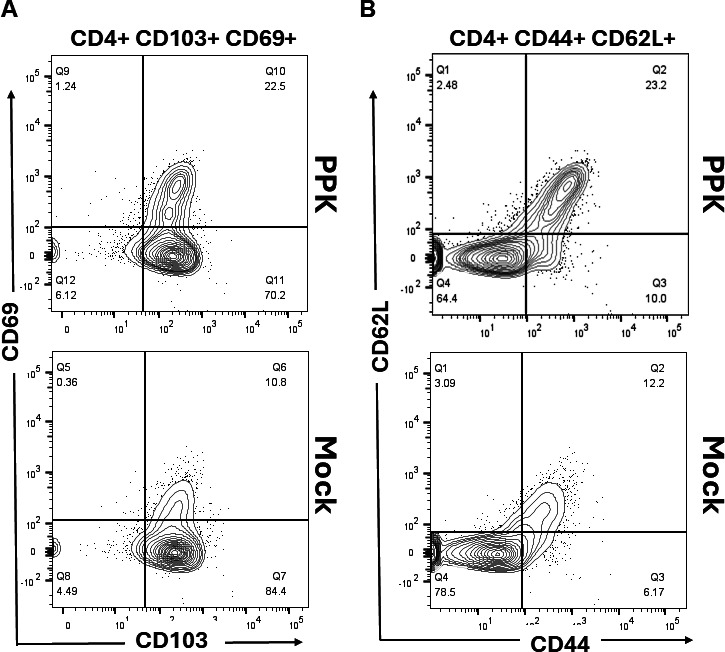
Increased frequencies of CD4^+^ memory T-cell population observed in response to prime-pull-keep vaccination approach. (A and B) Representative flow cytometry data showing the frequencies of memory CD4^+^ T_CM_, CD4^+^ T_EM_, and CD4^+^ T_RM_ cell subsets detected in HSV-1-infected TG of PPK-vaccinated and Mock-vaccinated HLA Tg rabbits. To study the CD4^+^ T-cell memory response in the context of the PPK vaccination approach, multiple markers like CD44, CD69, CD62L, and CD103 were analyzed.

Since the PPK vaccine enhanced protection as measured by reduced viral loads and herpetic corneal eye diseases in HLA-transgenic rabbits following HSV-1 infection, we sought to determine whether this protection was associated with increased frequencies of resident memory CD8^+^ and CD4^+^ T cells in the TGs. Vaccinated/PPK-treated and Mock-vaccinated/untreated rabbits were euthanized on day 58 post-infection, and the frequencies of HSV-1 peptide-specific CD8^+^ T cells of the resident memory CD8^+^ T cells expressing CD103, CD69; effector memory CD8^+^ T cells expressing CD62L, and CD44 among total cells (i.e., effector memory [TEM], resident memory [TRM], and central memory [TCM]) were determined by FACS, as described in Materials and Methods. The data in Fig. 4A show overall that the most dominant epitopes triggered a higher number of HSV-1-specific CD8^+^ T cells in PPK-vaccinated rabbits compared to Mock-vaccinated rabbits. The absolute count of these HSV-1-specific CD8^+^ T cells in the TG shows that in PPK-vaccinated rabbits, there are 2 to 5 times higher numbers of the dominant HSV-1 specific CD8^+^ T-cell epitopes (gD_53-61_, gB_342-350_, VP11_702-710_, and UL9_196-204_) in the TG compared to mock rabbits (Fig. 4B). The analysis of HSV-specific memory CD8^+^ T cells showed a significant increase in memory CD8^+^ T cells expressing CD44, CD69, and CD62L in PPK-vaccinated rabbits (Fig. 4A and B). More interestingly, we found a significant increase in CD103^+^ CD8^+^ T cells, CD44^+^CD62L^−^CD8^+^ T cells, and CD44^+^CD62L^+^CD8^+^ T cells in rabbits immunized with the PPK vaccine (Fig. 5). In addition, the PPK-immunized rabbits also exhibited higher frequencies of CD4^+^CD44^+^CD62L^+^ and CD4^+^CD103^+^CD69^+^ memory T cells (Fig. 6). These results demonstrate that the PPK vaccine expressing CXCL11 chemokine and IL-2/IL-15 bolsters the functional HSV-specific CD8^+^ and CD4^+^ T cells in the cornea and TG of HLA-A*0201 Tg rabbits.

**Fig 7:**
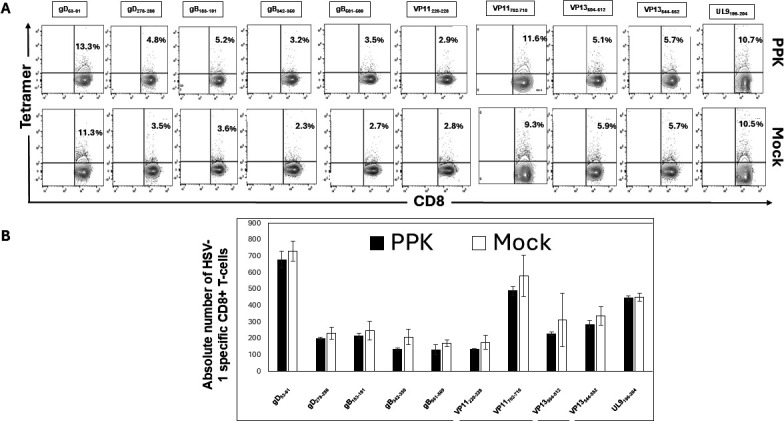
HSV-1-specific CD8^+^ T-cell frequencies in PPK-vaccinated HLA-A*0201 transgenic rabbits as observed in spleen. (A) Flow cytometry analysis of the frequencies of CD8^+^ T cells specific to HLA-A*0201-restricted HSV-1 epitopes (gD53-61, gD278-286, gB183-191, gB342-350, gB561-569, VP11220-228, VP11702-710, VP13504-512, VP13544-552, and UL9196-204) in spleen of PPK-vaccinated and Mock-vaccinated HLA-Tg rabbits. (B) The corresponding bar diagram shows an absolute number of HSV-1-specific CD8^+^ T cells in spleens of PPK-vaccinated and Mock-vaccinated HLA-Tg rabbits. All HSV-1-infected rabbits showed non-significant differences in HSV-1-specific CD8^+^ T-cell frequencies in the spleen at day 58 post-HSV-1 infection, which is also 28 days post-vaccination with PPK or empty vector. There was no significant difference between the two groups regarding the frequency of HSV-1 epitope-specific CD8^+^ T cells in the spleen (*P* > 0.05). The data are representative of two independent experiments, and the graphed values and bars represent the SD between the two experiments.

**Fig 8:**
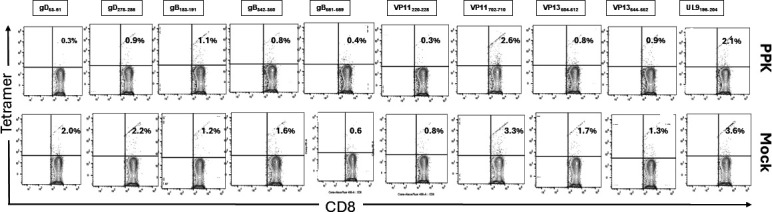
HSV-1-specific CD8^+^ T-cell frequencies in PPK-vaccinated HLA-A*0201 transgenic rabbits as observed in blood. (A) Flow cytometry analysis of the frequencies of CD8^+^ T cells specific to HLA-A*0201-restricted HSV-1 epitopes (gD53-61, gD278-286, gB183-191, gB342-350, gB561-569, VP11220-228, VP11702-710, VP13504-512, VP13544-552, and UL9196-204) in the blood of PPK-vaccinated and Mock-vaccinated HLA-Tg rabbits. All HSV-1-infected rabbits showed non-significant differences in HSV-1-specific CD8^+^ T-cell frequencies in the blood at day 58 post-HSV-1 infection, which is also 28 days post-vaccination with PPK or empty vector. There was no significant difference between the two groups regarding the frequency of HSV-1 epitope-specific CD8^+^ T cells in the blood (*P* > 0.05). The data are representative of two independent experiments.

The frequency of HSV-1-specific CD8^+^ T cells in the spleen as well as in the blood after vaccination shows that both the rabbit groups show no significant difference (Fig. 7 and 8). This demonstrates that the neurotropic AAV-8-based PPK vaccine is oriented toward the immune response to the sites of HSV-1 infection in the TG and cornea. Altogether, these results (i) indicate that immunization with mixtures of HSV-1-specific peptides (Table 1) combined with CXCL11 and IL-2/IL-15 treatment reduced ocular HSV-1 load, and decreased ocular herpetic disease; (ii) suggest that bolstering the number and function of HSV-specific CD8^+^ T cells that infiltrate the cornea and TG through a prime/pull/keep vaccine improved protection against ocular herpes infection and disease; and (iii) support HLA-A*02:01 Tg rabbits as a useful animal model for investigating the underlying mechanisms by which CD8^+^ T cells, specific to human HSV-1 CD8^+^ T-cell epitopes, mediate control of ocular herpes infection and disease.

## DISCUSSION

In the present study, we designed a multi-epitope prime/pull/keep HSV-1 vaccine candidate based on 10 asymptomatic (ASYMP) CD8^+^ T-cell peptide epitopes and 3 CD4^+^ T-cell epitopes selected from the HSV-1 glycoproteins D and B (gD and gB), viral tegument proteins (VP11/12 and VP13/14), and the DNA replication binding helicase (UL9), all preferentially recognized by CD8^+^ and CD4^+^ T cells from “naturally protected” HSV-1-seropositive healthy ASYMP individuals (who never had recurrent corneal herpetic disease). These asymptomatic human CD4+ and CD8^+^ T-cell epitopes were used as priming of HSV-1-specific CD4^+^ and CD8^+^ cells, CXCL11 for pulling HSV-1-specific CD4^+^ and CD8^+^ T cells to the site of infection (TG and cornea) and IL2 and IL15 to keep these HSV-1-specific CD4^+^ and CD8^+^ T cells in the site of HSV-1 acute and latent infection (TG and cornea) for longer time (Fig. 1). In preparation for the HLA transgenic rabbit experiments reported in this manuscript, we have used an HLA transgenic mouse model of ocular herpes to perform all the controls, including a side-by-side comparison of each component of the PPK vaccine alone or in combination (i.e., prime alone, pull alone, keep alone, PP together, PK together, or PPK). In those HLA transgenic mouse experiments, the combination of AAV8-CD4-CD8 epitopes-CXCL-11-IL-2 and AAV8-CD4-CD8 epitopes-CXCL-11-IL-15 prime/pull/keep vaccine gives the best protection against recurrent eye disease in the UV-B-treated mouse model. The data presented in this HLA transgenic mice study clearly show that frequent and functional HSV-1-specific CD8^+^ and CD4^+^ T_RM_ cells resided in the TG and cornea for at least 28 days post-vaccination in the PPK-vaccinated animals. The above results in the HLA transgenic mouse model of ocular herpes will be the subject of a future report. In this manuscript, we showed that immunization with a mixture of our PPK vaccine elicited polyfunctional CD8^+^ T-cell responses in HLA Tg rabbits that were associated with protection from recurrent ocular herpes infection and disease. Moreover, we observed that immunized HLA Tg rabbits developed frequent and functional HSV-specific CD69^+^CD103^+^CD8^+^ cytotoxic CD8^+^ T cells in the TG and cornea. These potent tissue-resident CD8^+^ T cells protected HLA Tg rabbits from virus reactivation in TG and replication in the eye and ocular herpetic disease in ocularly challenged rabbits with HSV-1 (McKrae). The results of this pre-clinical trial are as follows: (i) support the implementation of a prime/pull/keep ASYMP peptides-based vaccine strategy to strengthen the protective efficacy of tissue-resident CD8^+^ T cells against ocular herpes and (ii) indicate that ASYMP CD8^+^ T-cell epitopes selected from the HSV-1 glycoproteins D and B (gD and gB), viral tegument proteins (VP11/12 and VP13/14), and the DNA replication-binding helicase (UL9) proteins are more suitable candidates to be included in the next generation of ocular herpes prime/pull/keep peptide-based vaccines.

Complications from the HSV-1 infection range from mild symptoms, such as cold sores and genital lesions, to more serious complications, such as permanent brain damage from encephalitis in adults and neonates and blinding corneal inflammation (3, 5). Ocular HSV-1 infection is the leading cause of viral-induced corneal blindness in industrialized countries. Changes in sexual behavior among young adults have been associated with a recent increase in genital HSV-1 infection, resulting from oral-genital rather than genital-genital contact. Approximately 450,000 adults in the United States have a history of recurrent herpetic ocular disease (symptomatic; SYMP individuals), with approximately 20,000 individuals per year experiencing recurrent and potentially blinding ocular herpetic lesions (3, 4, 6, 7). The seropositive SYMP and ASYMP individuals are different with regard to CD8^+^ T-cell epitope specificity, the magnitude, and the phenotype of HSV-specific CD8^+^ T cells (3–7, 19, 30, 39). Thus, a vaccine that converts the presumably non-protective profile of CD8^+^ T cells seen in SYMP patients into the protective profile seen in ASYMP individuals will likely lead to a decrease in ocular herpes. Traditional vaccine formulations using native or recombinant proteins are generally ineffective in the induction of CD8^+^ T-cell responses (32). Clinical trials of HSV vaccines using selected HSV proteins mixed with adjuvant have shown limited efficacy in seronegative women, but not in men (reviewed in reference 3). This limitation results from the basic biology of Ag processing and presentation of epitopes to CD8^+^ T cells, necessitating the endogenous synthesis and presentation of HLA class I molecules. By contrast, our proposed PPK vaccine containing a mixture of the CD4^+^ and CD8^+^ T-cell epitopes, CXCL11 and IL2/IL15 induced strong CD8^+^ T-cell responses. In the present study, we demonstrated that immunization with mixtures of peptide vaccines exclusively bearing human epitopes from the HSV-1 glycoproteins D and B (gD and gB), viral tegument proteins (VP11/12 and VP13/14) and the DNA replication binding helicase (UL9) proteins that are mainly recognized by CD8^+^ T cells from HSV-1 seropositive healthy ASYMP individuals and CXCL11 and IL2 and IL15 (1) reduced infectious virus in tears and lessened ocular herpes following ocular challenge in therapeutically immunized HLA Tg rabbits. This peptide vaccine excludes SYMP epitopes that are recognized mostly by CD8^+^ T cells from SYMP individuals with a history of numerous episodes of recurrent ocular herpes disease. We have shown that these ASYMP epitopes in tandem with CXCL-11 and IL1/IL15, PPK vaccine given therapeutically to latently infected HLA Tg rabbits: (i) significantly decrease virus reactivation from TG (virus shedding in tears) and/or recurrent ocular disease and (ii) increase the numbers and functions of local HSV 1 glycoproteins D and B (gD and gB), viral tegument proteins (VP11/12 and VP13/14) and the DNA replication-binding helicase (UL9) epitopes specific CD8^+^ T cells over the existing immune response induced by the primary infection.

Recombinant non-replicating AAV-8 vector has been shown in many studies, including clinical trials, to be safe in human and animal models used topically to the cornea (40, 41). To confirm what has been already demonstrated, we have ocularly administered the empty AAV8 vector to non-infected rabbits and followed them for 28 days for eye disease, and data show that none of the rabbits have developed any kind of corneal disease (data not shown). It is likely that the AAV by itself induced AAV-specific CD4^+^ and CD8^+^ T_RM_ cells that infiltrate the TG and cornea of rabbit. However, the present study mainly focused on detecting frequencies and function of HSV-specific CD4^+^ and CD8^+^ T_RM_ cells that infiltrate the TG and cornea of HLA transgenic rabbit and their association with reduction of ocular herpes infection and disease in this HLA transgenic rabbit model of spontaneous ocular herpes. Using the HLA transgenic rabbit is the gold standard in those experiments because using HLA class I and HLA class II tetramers allows for specifically tracking HSV-specific CD4^+^ and CD8^+^ T_RM_ cells in vaccinated vs. mock-vaccinated animals

Both rabbit and mouse ocular herpes models have been successful for studying ocular HSV-1 infection and immunity, and each model resulted in new information and discoveries related to human HSV-1 ocular disease (reviewed in references 42 and 43). Mice have been the animal model of choice for most immunologists over the years, and results from mice have yielded remarkable insights into the role of CD8^+^ T cells in protection against primary herpes infection (7, 19, 44–47). Unfortunately, spontaneous reactivation of HSV-1 in mice is extremely rare, so the relevance of these findings to *in vivo* HSV-1 spontaneous reactivation cannot be determined in mice (48). The rabbit ocular herpes model has been especially important for investigating viral reactivation and recurrent ocular disease (42, 43, 49). Unlike mouse eyes, but similar to human eyes, the surfaces of the rabbit eyes are relatively immunologically isolated from systemic immune responses (35, 42, 50). Using the “humanized” HLA transgenic rabbit model of ocular HSV-1 that mounts “human-like” CD8^+^ T-cell immune responses (HLA Tg rabbits), we found that immunization of HLA Tg rabbits with the three human CD8^+^ T-cell epitopes induced strong CD8^+^ T cell-dependent protective immunity against ocular herpes infection and disease. From a practical standpoint, the size of rabbit corneas is significantly larger than those of mice and offers a plentiful amount of tissues for phenotypical and functional characterization of HSV-specific T cells using individual tissues (19, 29, 35, 42, 50). To overcome the hurdle of rabbits that do not mount T-cell responses specific to human HLA-restricted human epitopes, we introduced the “humanized” HLA Tg rabbit model of ocular herpes whereby the rabbits express human leukocyte antigen (HLA class I molecules) (30, 51). Ocularly infected HLA Tg rabbit mounted HLA-A*0201-restricted CD8^+^ T-cell responses to HSV-1 glycoproteins D and B (gD and gB), viral tegument proteins (VP11/12 and VP13/14) and the DNA replication binding helicase (UL9) epitopes similar to that from HLA-A*0201-positive HSV seropositive humans. Since the expression of the rabbits’ own MHC class I molecules might interfere with the human HLA-A*0201-restricted responses (9), and to ensure that all rabbits had a high level of expression of HLA-A*0201 molecules in over 90% of their CD8^+^ T cells, only those HLA Tg rabbits with these characteristics were used in this investigation. These results confirm our previous report that all gD epitopes recognized by CD8^+^ T cells from HSV-1 infected HLA Tg rabbits are recognized by CD8^+^ T cells from HLA-A*0201-positive HSV seropositive humans (30). Thus, the HLA Tg rabbit is a useful model for pre-clinical testing of candidate vaccines bearing human T-cell epitopes. The HLA Tg rabbit model allows testing the hypothesis that a vaccine that induces appropriate human T-cell responses to HSV-1 can decrease HSV-induced ocular disease. These findings should guide the development of a safe and effective T-cell-based herpes vaccine. Similarly, to the rationale of choosing the rabbit animal model to study the protective efficacy of PPK against recurrent herpetic disease, we have chosen the McKrae strain over other HSV-1 strains that have been shown to need corneal scarification and have poor reactivation phenotype and recurrent corneal disease in mouse and rabbit models such as (KOS strain) (37). In conclusion, four principal findings were determined in the pre-clinical results obtained with HSV-1 infected HLA Tg rabbits in this report. First, a human herpes vaccine that exclusively contains a mixture of human ASYMP CD8^+^ T-cell epitopes derived from the HSV-1 glycoproteins D and B (gD and gB), viral tegument proteins (VP11/12 and VP13/14), and the DNA replication binding helicase (UL9) proteins along with CXCL11, IL2, and IL15 provides protection in HLA-A*0201 Tg rabbits against ocular herpes infection and disease. Second, frequent polyfunctional HSV-1 ASYMP epitope-specific CD8^+^ T-cells were induced by the ASYMP human epitopes from gB, gD, VP11/12, and UL-9 proteins and correlated with protection against ocular herpes infection and disease in HLA Tg rabbits, following HSV-1 ocular challenge. Third, the results demonstrate for the first time that the IL2 and IL15 axis is of paramount importance in keeping the protective CD8^+^ T cells to the TG and corneal tissues associated with clearance of ocular herpes infection and disease for a longer period. Fourth, the study validates the HLA-A*0201 Tg rabbit model of ocular herpes for pre-clinical testing of future herpes vaccine candidates bearing human ASYMP CD8^+^ T-cell epitopes against ocular herpes. Overall, the pre-clinical results determined in HLA Tg rabbits draw attention to the prime/pull/keep vaccine strategy, as an alternative to currently used protein-based vaccines, to strengthen the protective efficacy of tissue-resident CD8^+^ T cells against ocular herpes infection and disease.

## Data Availability

The gene IDs and nucleotide sequence accession numbers associated with HSV-1 proteins mentioned in this article are as follows: HSV-1 glycoprotein B (gB) (gene ID 330109, accession number M21629.1); glycoprotein D (gD) (gene ID L09243.1, accession number AAA19629.1); viral tegument protein VP11/12 (UL46) (gene ID 2703413, accession number NC_001806.2); viral tegument protein VP13/14 (UL47) (gene ID 2703415, accession number NC_001806.2); and DNA replication binding helicase protein (UL9) (gene ID 2703434, accession number NC_001806.2).
